# Eccentric versus traditional resistance exercise for older adult fallers in the community: a randomized trial within a multi-component fall reduction program

**DOI:** 10.1186/s12877-017-0539-8

**Published:** 2017-07-17

**Authors:** Paul LaStayo, Robin Marcus, Leland Dibble, Bob Wong, Ginette Pepper

**Affiliations:** 1 Department of Physical Therapy and Athletic Training, University of Utah, 520 Wakara Way, Salt Lake City, UT 84109 USA; 2 College of Nursing, University of Utah, Salt Lake City, UT USA

**Keywords:** Falls, Exercise, Eccentric, Aging, Prevention

## Abstract

**Background:**

Addressing muscle deficits within a multi-component exercise fall reduction program is a priority, especially for the highest risk older adults, i.e., those who have fallen previously. Eccentric resistance exercise with its high-force producing potential, at a low energetic cost, may be ideally-suited to address muscle impairments in this population. The purpose of this study was to compare the effects of resistance exercise via negative, eccentrically-induced, work (RENEW) versus traditional (TRAD) resistance exercise on mobility, balance confidence, muscle power and cross sectional area, as well as the number of days high fall risk older adults survived without a fall event over a 1 year period.

**Methods:**

Randomized, two group, four time point (over 1 year) clinical trial testing RENEW versus TRAD as part of a 3 month multi-component exercise fall reduction program (MCEFRP). Primary outcomes of mobility, balance confidence, muscle power output and cross sectional area were analyzed using mixed effects modeling. The secondary outcomes of days to fall and days to near-fall were analyzed using survival analysis.

**Results:**

The MCEFRP did have an effect on fall risk factors considered reversible with exercise interventions though there was no differential effect of RENEW versus TRAD (*p* = 0.896) on mobility, balance confidence, muscle power and cross sectional area. There were also no group differences in the number of days survived without a fall (*p* = 0.565) or near-fall (*p* = 0.678). Despite 100% of participants having at least one fall in the year prior to the MCEFRP, however, after 3 months of exercise and 9 months of follow-up <50% had experienced a fall or near fall.

**Conclusions:**

There were no differential effects of RENEW or TRAD as components of a MCEFRP on the primary or secondary outcomes. The two modes of resistance exercise had identical effects on fall risk and fall-free survival.

**Trial registration:**

NCT01080196; March 2, 2010 (retrospectively registered).

## Background

Older adults are more likely than other age groups to suffer serious injury from a fall and more than 40% of community dwelling adults over 65 years of age fall each year, making falls the leading cause of injury-related death [1]. Fall incidence doubles in those beyond 75 years of age [2, 3] and a previous history of falling is an additive risk for future falls [3, 4]. Muscle impairments in the lower extremities have been identified as critical, yet modifiable, risk factors that should be therapeutically targeted in exercise interventions for fall prevention [5]. Specifically, muscle weakness imposes a three to four-fold greater risk of a fall. Muscle atrophy too has an impact on fall risk though lesser than muscle weakness [6, 7]. Deficits in muscle power are even more important than muscle strength for safe mobility function through dynamic balance and protective responses [8, 9]. This focus on muscle for fall prevention has been highlighted in The National Council on Aging’s “Falls Free Initiative” [10] and in consensus opinions of the Centers for Disease Control and Prevention [11] and clinical guideline statements from the Academy of Geriatric Physical Therapy and the American Physical Therapy Association [12].

Resistance exercise can address deficits in muscle power and size along with impairments in mobility and confidence. Therefore, resistance exercise has been systematically highlighted as a necessary component of a successful multi-component exercise fall reduction program (MCEFRP). Eccentric resistance exercise has gained considerable attention within the last 15 years due to its appropriateness and tolerability for older adults who might otherwise be limited by their diminished muscular strength and aerobic capacity [13, 14]. The unique properties of eccentric muscle action (low energy cost, high muscle force production), coupled with findings from previous pilot trials [15–19] that eccentric exercise can mitigate deficits in muscle size, strength and mobility while lowering the fall risk profile of older adults, makes eccentric exercise an alluring add-on to a MCEFRP. Further, the amplified effects of eccentric versus concentric exercise on muscle strength and mass have been systematically reported in healthy adults [20]. To date, however, no large-scale randomized controlled trial has compared the relative merits of resistance exercise via negative, eccentrically- induced, work (RENEW) verses traditional resistance exercise (TRAD) as part of a MCEFRP on mitigating fall events in a high fall-risk older adult population.

The purpose of this randomized clinical trial was to test RENEW versus TRAD as the resistance exercise component part of a 3 month MCEFRP. Importantly, this trial was not designed to test whether a MCEFRP was effective at mitigating falls as multiple studies have reported reductions of ~13–30% and these studies have been recently reviewed systematically [21–23]. Here we simply were testing the merits of RENEW as the resistance exercise component part of a MCEFRP and comparing it to a TRAD component. We hypothesized the effect of RENEW as part of the MCEFRP would be greater than the effect of TRAD on increasing mobility and balance confidence, muscle power, and muscle lean tissue cross sectional area. Further, we expected those experiencing RENEW, versus TRAD, would increase the number of days high fall risk older adults survived without a fall event over a 1 year period.

## Methods

### Participants, randomization and study time points

**Fig. 1:**
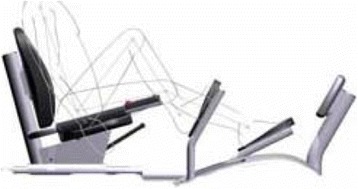
Eccentric ergometer used by the RENEW group. Participants resisted the alternating motor-driven movement of the pedals towards them. The force exerted by the ergometer motor exceeds the force of the participant creating eccentric or negative work of the knee and hip extensor muscles

Following a multi-step screening process that included contacting potential participants from a patient database at the University of Utah Health Sciences Center via mailed letters, follow-up phone screening, and finally an in-clinic assessment, 134 older adults met the criteria for high fall risk and volunteered to participate (see Table 1 and Fig. 1). All participants agreed to engage in a three-month (36 sessions of 1 h) multi-component exercise fall reduction program (MCEFRP) that included either a traditional (TRAD) or eccentric (RENEW) resistance-training program for the legs.

**Table 1:** Resistance exercise training schedule for TRAD group

Training week	Training duration (sets & reps)	Lower extremity exercises
1	3 × 15	Leg Press Machine (60%–65% of 1RM) Standing 4 directional straight leg raises
3–12	3 × 15	Leg Press Machine (70% of 1 RM) Standing 4 directional straight leg raises

Participants were deemed eligible if: at least 65 years of age or older and had experienced at least one fall in the previous 12 months. They needed to be: community dwelling and ambulatory with a gait speed ranging from of 0.42 to 1.3 m*s^−1^, able to recall all three items (or one to two items with a normal clock drawing test) on the Mini-Cog™ instrument for dementia screening [24], managing two or more co-morbid conditions- though cleared by their physician to participate in a 60 min (with rests) MCERFP. Potential participants were deemed ineligible if: they had a progressive diagnosed neurologic disease (e.g., Parkinson’s, multiple sclerosis, Guillain-Barre, Alzheimers), any dystrophies or rheumatologic conditions that primarily affects muscle (e.g., muscular dystrophy, polymyalgia rheumatica), already participated in a MCEFRP or if they were currently performing (or had performed) regular (3×/week) aerobic (defined as hiking, fast-walking, jogging, running swimming or cycling) or resistance (defined as weight training with bands, cable, free-weights or weight-machines) exercise over the past 12 months. Individuals were also excluded if they had any of the absolute contraindications for a magnetic resonance imaging scan.

A randomization process with blocks of ten (5 TRAD and 5 RENEW) insured equivalency in the number of subjects and the same proportion of men and women were assigned into each of the two groups. The participants were assessed over a one-year period via four study time points: pre-MCEFRP (0 months); post-MCEFRP (3 months); and nine (9 months) and twelve (12 months) follow-up months after enrollment. A statement regarding the study’s purpose, processes, procedures, benefits and risks were presented to each subject in both a written (informed consent document) and verbal format. All of the subject’s questions (if any) were addressed and each subject signed an informed consent document approved by the Institutional Review Board at the University of Utah. This clinical trial (NCT01080196) was retrospectively registered on March 2, 2010.

### MCEFRP intervention with RENEW or TRAD resistance exercise of the legs

Participants trained for 60 min per session, three times per week for 3 months as part of the MCEFRP that included resistance training of the lower extremities. Training sessions were individualized and supervised, but performed in groups of two to five participants over a 3 h period at a rehabilitation wellness center at the University of Utah. The MCEFRP consisted of multiple modes of exercises performed in a circuit that alternated higher-intensity and dynamic activities with lower-intensity, static tasks. Aerobic exercise was performed on a NuStep recumbent trainer (NuStep Inc., Ann Arbor, MI), seated stationary cycle ergometer, or overground treadmill. Flexibility exercises [25] designed to improve joint range of motion were completed for 30–60s per exercise at a perceived intensity of a strong pulling sensation without pain included: pectoralis stretching in a doorway, seated hamstrings stretching, standing calf stretching, trunk rotation stretches, and prone positioning for hip flexor stretching. Balance exercises and challenges over a 15–20 min period were individually adjusted and progressed [26, 27] included both static and dynamic base of support regimens and incorporated varied head positions and eyes open or closed for altered vestibular and visual sensory stimulation. Upper extremity resistance exercises [28] of the shoulder rotator cuff, deltoid and scapular muscle systems in addition to the elbow flexors, extensor and rotators were performed over a 15–20 min period using free weights. The free weights for these upper extremity resistance exercises were increased as tolerated every 2 weeks provided the participants could complete three sets of 15 repetitions with appropriate form. The only difference in the MCEFRP for the two groups was the type of lower extremity resistance training performed, i.e., RENEW or TRAD. There was no attempt at matching the workloads of the two lower extremity resistance exercise regimens, though the amount of time spent doing lower extremity resistance exercise progressed in both groups to a maximum of 15 min. Adhering to a minimum of 18 of the 36 MCEFRP sessions was required.

The TRAD resistance exercise group performed (Table 1) three sets of 15 repetitions of a seated bilateral leg press exercise (Tuff Stuff PS −230 Deluxe Leg Press, Tuffstuff, Chino, CA) at 60–65% of their one repetition maximum (RM) for the initial 2 weeks. Training sessions for the remaining 10 weeks were performed at 70% of 1-RM, which was assessed every 2 weeks thereafter. In addition, the TRAD group performed standing multi-directional straight leg exercises with a weighted cuff placed just proximal to the ankle. The training loads for this exercise were increased as tolerated every 2 weeks provided the participants could complete three sets of 15 repetitions with appropriate form.

**Fig. 2:**
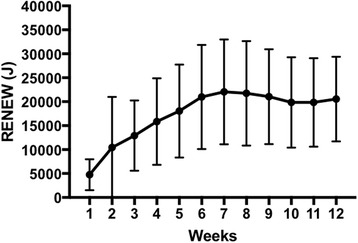
Mean RENEW (J) per week over the 12 weeks of the MCEFRP

The RENEW group performed (Table 2 and Fig. 1) progressive resistive eccentric exercise of the knee and hip extensor muscles using a recumbent stepper-ergometer (Eccentron™, Baltimore Therapeutic Equipment, Hanover, MD) as described previously [19]. Briefly, the stepper speed ranged between 12 and 18 rpm as the participant resisted the stepper pedal action and eccentric muscle contractions were induced in the knee and hip extensor muscles. Visual feedback of the work performed for each revolution was displayed on a computer monitor. Participants performed eccentric resistance exercise and negative work (Fig. 2) from approximately 15–75 degrees of knee flexion as they resisted the motorized movement of the stepper pedals via resistance action of the knee and hip extensors. Perceived exertion was assessed with the Borg rating scale [29] between 6 and 20 (Table 2). In the first and second week of RENEW, sessions lasted three to 5 min and were performed at a “very, very light” intensity (Borg rating = 7) and “very light” (Borg rating = 9) while resisting the stepper pedal action. During subsequent weekly training sessions of 5–12 min, participants were gradually allowed to resist the pedal action with more exertion as they progressed from a “fairly light” intensity (Borg rating = 11) at weeks three to four to a “somewhat hard” intensity level (Borg rating = 12–13) at weeks 5–12. The duration of each session was progressively increased to a maximum 15 min duration of RENEW during weeks 5–12.

**Table 2:** Resistance exercise training schedule for RENEW group utilizing eccentric ergometer

Training week	Training duration (min)	Rate of perceived exertion
1	3–5	7 (very, very light)
2	5	9 (very light)
3	5–10	11 (fairly light)
4	10–12	11–13 (fairly light to somewhat hard)
5–12	12–15	11–13 (fairly light to somewhat hard)

### Mobility

The 6 min walk (6 MW) test, a measure of the distance (m) a subject walks in 6 min, was used to assess overall mobility [30]. Self-selected gait-speed was measured over a 50-ft course. Individuals were instructed to walk at a comfortable pace starting at the word “go.” They were asked to walk out 25-ft and back. Timing took place from the command “go” until the starting line was crossed on the way back. Participants were allowed to use any walking aid they used on a daily basis. The 6 MW test has high test-retest reliability in older populations with various co-morbid conditions [31].

### Balance confidence

Self-reported level of balance confidence was assessed with the Activities Specific Balance Confidence (ABC) Scale. The ABC Scale is able to discriminate between fearful and non-fearful subjects and between those who avoid activity due to fear of falling [32]. This 16-item questionnaire asks participants to score their level of confidence in performing situation-specific activities such as “reaching at eye level,” “reaching on tiptoes,” “picking up slipper from floor,” and “walking in crowded mall” “without losing . . . balance or becoming unsteady.” Each item is scored from 0 to 100%, with 0% being no confidence and 100% being full confidence in the ability to perform the activity without losing balance. The total ABC Scale score is the average sum of the individual item scores. The ABC Scale yields data with strong test-retest reliability (*r* = .92) and responsiveness when used with community dwelling elderly adults aged 65–95 years [32, 33].

### Leg extensor muscle power

Leg extension power (watts) of each leg individually was measured on a Nottingham power rig (Medical Engineering Unit, University of Nottingham Medical School, Nottingham, UK). Participants were seated in an upright position with arms folded. The seat was adjusted until comfortable extension of the knee with full depression of the foot pedal was reached. Participants right and left legs were tested individually though the average single leg power of was used for analysis. Participants were instructed to depress the foot pedal as hard and quickly as possible. After three warm-up trials at 50%, 75%, and 100% effort, six test trials per leg were performed and the average of the three highest trials per leg were recorded. The leg extension power rig is a valid, reliable and feasible means of assessing leg extension muscle power across the lifespan in both males and females [34].

### Thigh muscle lean tissue cross sectional area

Magnetic resonance imaging (MRI) was used for determination of the cross-sectional area (cm^2^) of lean muscle mass as previously described [16, 35]. Bilateral MRI scans of the thighs were obtained and subjects were placed supine in a 3.0 Tesla whole body imager (Siemens Trio, Siemens Medical, Erlangen, Germany). The legs were scanned in a coronal plane and the midpoint of the thigh was determined and defined as halfway between the superior margin of the femoral head and the inferior margin of the femoral condyles. Axial imaging (5 mm thick slices at 1 cm intervals) of the legs was then performed over 1/2 the length of the femur, centered at the midpoint of the thigh. Separate fat and water images were created with custom software using the three-point Dixon method. A tissue model was then used to calculate estimates of total fat and non-fat volume fractions on a per-pixel basis, which were displayed in image form. Five images from the middle 1/3 of each thigh were used to determine average cross-sectional area of lean tissue. Manual tracing eliminated subcutaneous fat and bone and isolated the fascial border of the thigh to create a subfascial region of interest. Total lean tissue was calculated by summing the value of percent lean tissue fraction over all pixels using custom-written image analysis software (MATLAB; The MathWorks, Natick, Massachusetts). This sum was multiplied by the area of each pixel to give total lean tissue cross-sectional area within the region of interest. The same investigator, blinded to time point of the scan and slice location, performed measurements of individual participants at each time point. Intra-investigator reliability of this technique in our laboratory is excellent (mean intra-class correlation coefficient = 0.99) and has been previously published [16].

### Fall and near-fall events (days survived without a fall or near-fall)

A fall was defined for this study as unintentionally coming to rest on the ground, floor, or other lower level [36]. A near- fall occurred when the individual felt a fall was imminent, but was avoided by a compensatory action of the individual [37]. Fall events, consisting of falls and near-falls*,* were monitored for 1 year after enrollment in accordance with consensual recommendations for fall event monitoring [21]. Consistent with the consensus recommendations near-falls were also included to increase the sensitivity of measurement of the effects of fall prevention interventions [38]. During the 3 months (0–3 months) of the MCEFRP intervention project personnel instructed participants in fall event definitions and asked at each session (3 times per week) if they had a fall, near fall, new medication, or change in health status. If there was a report of a fall or near-fall the project staff recorded the event and reminded the participant to call the reporting number that had been provided on a refrigerator magnet as soon as possible after an event throughout the entire study. At the conclusion of the active intervention period (3 months), participants were provided nine stamped postcards for monthly (3 months – 12 months) reporting of falls or near-falls, changes in medication use, and changes in physical condition. A designated research assistant monitored the telephone report line and monthly postcards. If a postcard was not received for a month or an event that had not been reported on the telephone or via a returned postcard was identified, the research assistant initiated contact. During the telephone interview regarding fall or near-fall events regardless of how the event was detected, a structured interview about the circumstances and consequences of the fall or near-fall event was conducted. A proxy (family member or friend designated by the participant at enrollment) was contacted if the participant could not be reached after five attempts or the participant sounded confused or unreliable.

### Statistical analysis

An intent-to-treat approach was used and any missing primary or secondary data points were assumed to be missing at random. Primary outcomes (*mobility, balance confidence, leg extensor power output and thigh muscle lean tissue cross sectional area)* were analyzed using mixed effects modeling, conducted with MIXED procedure (SPSS, V21). Fixed effects included: time (pre-MCEFRP--0 months; post-MCEFRP--3 months; 6 months following the MCEFRP--9 months; and 9 months following the MCEFRP--12 months), group (RENEW vs. TRAD), and time X group interaction. Participants’ were added as a random effect. Secondary outcomes (days to fall, days to near-fall) were analyzed using survival analysis, conducted with Kaplan-Meier procedure (SPSS, V21). Estimated marginal means (± standard error or 95% confidence intervals) are reported below and in Table 4.

## Results

### Participants and MCEFRP adherence

**Fig. 3:**
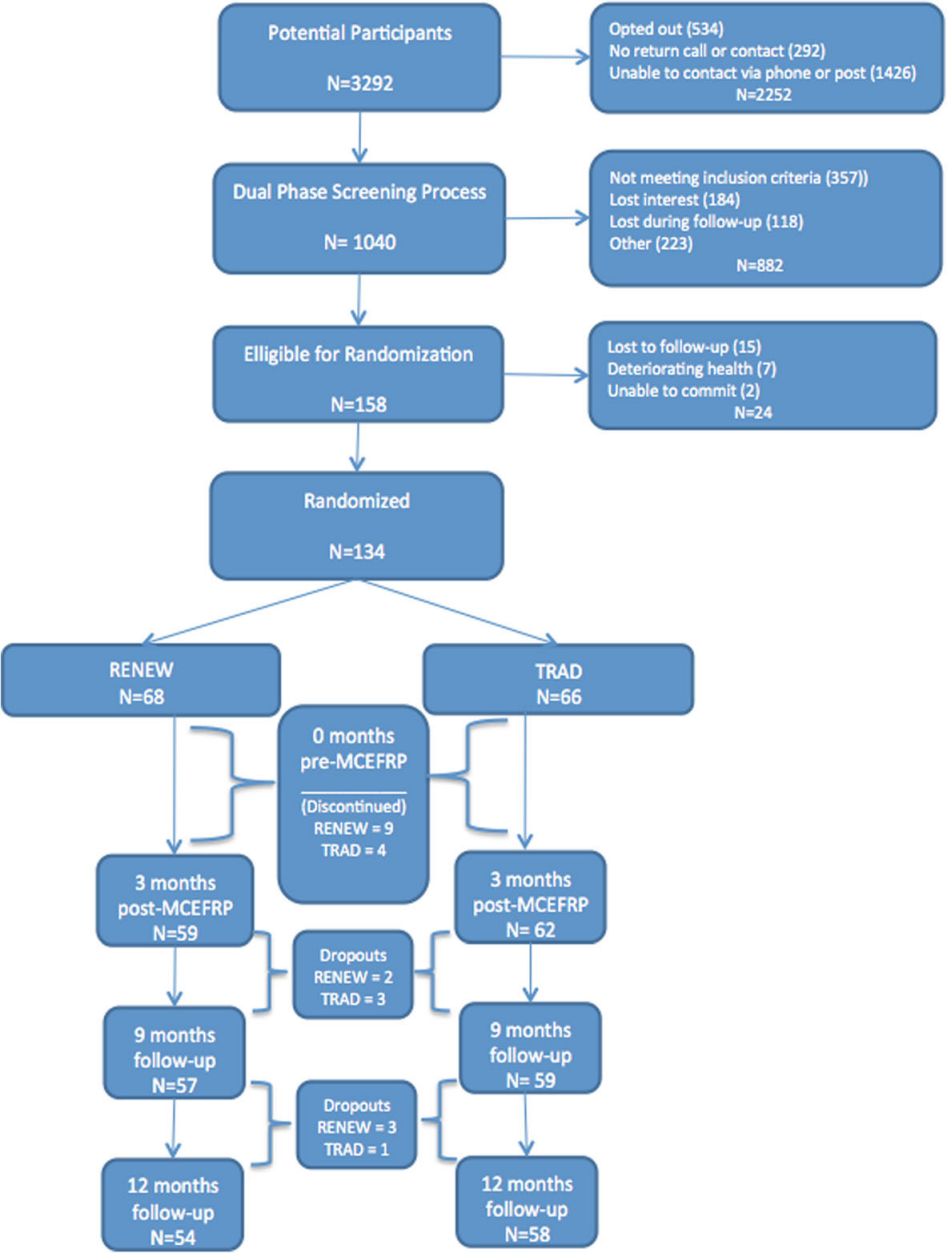
CONSORT diagram for study

Older adults who experienced one or more falls in the previous year, *n* = 134 (47 males, 87 females) with a mean age of 76.1 years (range 65–93 years); a BMI >25 kg*m^−2^ and managing more than five comorbid conditions met the criteria for inclusion as high fall risk and volunteered to participate in this 1 year trial. See Table 3 for participant characteristics. The randomization process resulted in 68 participants assigned to the RENEW group and 66 to TRAD group. The RENEW group had 13% of participants discontinue the MCEFRP and the TRAD group’s MCEFRP attrition was 6%. During the follow-up time points there were 5 and 4 dropouts for RENEW and TRAD respectively (see Fig. 3). All participants completed the requisite minimum 18 MCEFRP sessions and ≥90% adhered to at least 29 of the 36 exercise sessions.

**Table 3:** Participant characteristics

	RENEW (*n* = 68)		Traditional (*n* = 66)		
Characteristic	n or mean	% or SD	n or mean	% or SD	*p*-value
Age (years)	76.59	7.39	75.59	6.98	0.42
BMI	27.09	4.97	28.35	5.74	0.18
Comorbidities	5.18	2.26	5.20	2.36	0.95
Drugs	4.44	3.98	5.19	3.57	0.25
Gender					
Female	45	66.18	42	63.64	0.86
Male	23	33.82	24	36.36	
Hispanic ethnicity					0.24
Yes	0	0.00	2	3.03	
No	68	100.00	64	96.97	
Race					0.30
American Indian or Alaska Native					
Asian	1	1.47	0	0.00	
Black or African American	0	0.00	1	1.52	
White	67	98.53	63	95.45	
More than one race	0	0.00	1	1.52	
Unknown or not reported	0	0.00	1	1.52	
Smoker					1.00
No	67	98.53	66	100.00	
Yes	1	1.47	0	0.00	
Marital status					0.93
Divorced	9	13.24	9	13.64	
Married	39	57.35	39	59.09	
Single	4	5.88	5	7.58	
Widowed	16	23.53	13	19.70	
Education					0.26
0–8 years	2	2.99	0	0.00	
1–3 years of high school	2	2.99	1	1.52	
High school graduate/GED	4	5.97	7	10.61	
Some college/technical school	21	31.34	12	18.18	
Associate Degree	0	0.00	1	1.52	
Bachelor’s Degree	13	19.40	18	27.27	
Post-graduate education	25	37.31	27	40.91	
Employment status					0.87
Employed full-time	4	5.97	3	4.55	
Employed part-time	7	10.45	5	7.58	
Not employed outside the home	0	0.00	1	1.52	
Retired	56	83.58	57	86.36

### Mobility

There was no interaction effect, F (3, 319) = 0.618, *p* = 0.604, differential group differences over time, for the 6 MW test. See Table 4 with group mean estimates and 95% CI. A time effect, F (3, 319) = 4.10, *p* < 0.001, both exercise groups combined, occurred with an increase in distance walked in 6 min after the 3 month MCEFRP (0 months =407.297 ± 10.971; 3 months =424.53 ± 11.10). This increase was maintained at the 9 months (419.31 ± 11.21) and 12 months (420.16 ± 11.23) time points. There was no group effect, F (1, 190) = 0.017, *p* = 0.896.

**Table 4:** Group mean estimates and 95% CI of mobility, balance confidence, leg extensor muscle power, and thigh muscle lean tissue cross sectional area across the four time points

	0 months (pre- MCEFRP)		3 months (post- MCEFRP)		9 months		12 months	
Mean (95% CI)	RENEW	TRAD	RENEW	TRAD	RENEW	TRAD	RENEW	TRAD
Six Minute Walk Distance (m)	405.21 (367.40; 443.03)	416.13 (379.17; 453.10)	425.76 (386.06; 465.47)	439.06 (399.85; 478.26)	424.09 (380.26; 467.92)	449.10 (408.33; 489.86)	439.18 (394.65; 487.72)	438.15 (397.39; 478.92)
Activities Specific Balance Confidence (%)	73.03 (68.26; 77.81)	75.49 (70.77; 80.21)	82.47 (77.46; 87.47)	80.71 (75.83; 85.60)	80.11 (74.60; 85.63)	80.94 (75.74; 86.15)	82.25 (76.82; 87.68)	80.93 (75.73; 86.13)
Leg Extension Power (W)	88.84 (75.60; 102.08)	99.13 (86.04; 112.23)	106.55 (92.67; 120.44)	110.47 (96.92; 124.02)	113.66 (98.37; 128.95)	124.77 (110.53; 139.02)	104.73 (99.42; 129.55)	122.74 (108.30; 137.18)
Thigh Lean Tissue CSA (cm_2_)	93.38 (89.11; 97.64)	93.71 (89.41; 98.00)	94.20 (89.87; 98.54)	94.44 (90.16; 98.72)	93.00 (88.87; 97.35)	91.97 (87.67; 96.26)	92.60 (88.25; 96.95)	92.18 (87.88; 96.47)

*MCEFRP* multi-component exercise fall reduction program, *RENEW* resistance exercise via negative, eccentrically-induced, work, *TRAD* traditional resistance exercise, *CI* confidence interval, *CSA* cross sectional area

### Balance confidence

There was no interaction effect, F (3, 326) = 1.402, *p* = 0.242, differential group differences over time, on the ABC scale. See Table 4 with group mean estimates and 95% CI. A time effect, F (3, 325) = 14.153, *p* < 0.001, both exercise groups combined, occurred with an increase in balance confidence after the 3 month MCEFRP (0 months = 74.652 ± 1.389; 3 months = 81.040 ± 1.428). This increase was maintained at the 9 months (79.878 ± 1.466) and 12 months (80.576 ± 1.471) time points. There was no group effect, F (1, 142) = 1.996, *p* = 0.160.

### Leg extensor muscle power

There was no interaction effect, F (3, 334) = 0.672, *p* = 0.570, differential group differences over time, for the average (right and left leg) power produced during simultaneous knee and hip extension. See Table 4 with group mean estimates and 95% CI. A time effect, F(3, 334) = 33.446, *p* < 0.001), both exercise groups combined, occurred with an increase in leg extensor muscle power after the 3 month MCEFRP (0 months = 94.989 ± 3.867; 3 months = 107.928 ± 3.937). This increase was maintained at the 9 months (116.373 ± 3.991) and 12 months (116.496 ± 4.005) time points. There was no group effect, F (1, 164) = 2.206, *p* = 0.139.

### Thigh muscle lean tissue cross sectional area

There was no interaction effect, F (3, 286) = 1.006, *p* = 0.390, differential group differences over time, for the average (right and left leg) cross sectional area of lean tissue. See Table 4 with group mean estimates and 95% CI. A time effect, F (3, 286) = 8.595, *p* < 0.001), both exercise groups combined, occurred with a decrease in lean tissue at the 9 month and 12 month time points; 0 month (93.542 ± 1.761), 3 month (94.322 ± 1.767), 9 month (92.481 ± 1.771), and 12 month (92.386 ± 1.772). There was no group effect, F (1, 343) = 0.008, *p* = 0.930.

### Fall and near-fall events (days to fall or near-fall and # of individuals who had an event)

**Fig. 4:**
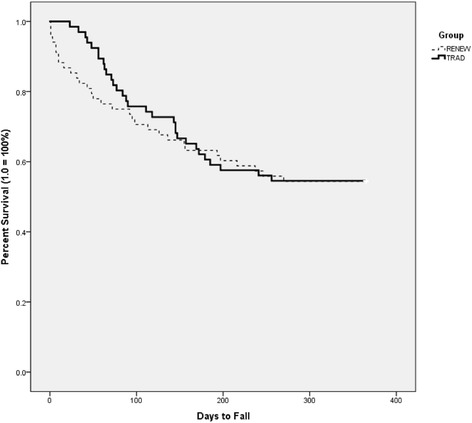
Survival Curve (Kaplan Meier) of number of days high fall risk older adults survived without a fall over a one year period

**Fig. 5:**
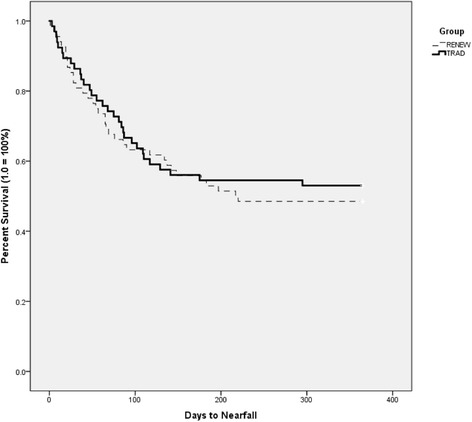
Survival Curve (Kaplan Meier) of number of days high fall risk older adults survived without a near fall over a one year period

There were no group differences in the number of days survived without fall event, i.e., a fall (RENEW = 239.00 ± 18.00; TRAD = 249.67 ± 16.38; c^2^ (1) = 0.332 (Breslow test), *p* = 0.565) or near-fall (RENEW = 216.35 ± 18.38; TRAD = 226.92 ± 18.76; c^2^(1) = 0.172, *p* = 0.678). See Figs. 4 and 5 for Kaplan Meier survival curves. Overall 61 participants (46%) had at least one fall (range of falls per participant = 1–15) and 66 participants (49%) had at least one near-fall (range of near-fall per participant = 1–26) over the 12 months.

## Discussion

During this 1 year randomized clinical trial with high fall risk older adults there were no differential effects of RENEW or TRAD as components of a MCEFRP on the fall risk variables of mobility, balance confidence, muscle power, nor muscle lean tissue cross sectional area. Further, there was no differential effect of RENEW or TRAD on the number of days high fall risk older adults survived without a fall or a near-fall event. These results were not expected since earlier findings with mobility-limited [16, 18, 19], frail [15] and generally healthy [39, 40] older adults engaging in eccentric resistance exercise of the lower extremities for 10–12 weeks have previously demonstrated improved mobility and muscle responses that exceeded those stemming from more traditional-types of resistance exercise regimens. Two defining properties of eccentric muscle contractions, i.e., the force production potential being uniquely high yet the energy cost to produce this force being uniquely low [41–43], are thought to underlie these previous RENEW effects. The characteristics of eccentric resistance exercise interventions have recently been reviewed and deemed well-suited for older adult rehabilitation populations [44] since many are exercise-limited and impaired in their abilities to produce sufficient muscle force to preserve their muscle mass and function. Moreover, eccentric muscle control is an essential component of balance recovery [45, 46]. Without sufficient load on lower extremity muscles, many older adults enter a downward spiral of sarcopenia, which can result in life-threatening falls [47–49]. Despite the potential for amplified muscle, balance and confidence responses after eccentric exercise others [50–52] have reported that either eccentric or traditional resistance exercise-induced loads on muscle can produce similar results in older adults. This study is the first to report the effect of RENEW as part of a MCEFRP on the prevention of fall events. We hypothesized more fall-event-free survival days would follow in the RENEW group over 1 year, but instead saw an equivalent effect to the TRAD group in the number of days a fall or near fall event were averted.

A recent updated review [22] of the literature [21] reinforces the notion that multi-component exercise interventions can reduce the risk and rate of falls in at-risk older adults living in the community. Exercise programs of three or more hours per week that include challenges to balance can reduce the rate of falls by 39% [23, 53]. Moreover, the evidence supports the hypothesis that increasing muscle strength and power should reduce the risk of falls [5]. The MCEFRP did have an effect on fall risk factors considered reversible with exercise interventions. That is, independent of whether RENEW or TRAD was employed modest increases in mobility (4%), balance confidence (6%) and leg muscle power (14%) did occur after engaging in a MCEFRP and these improvements were sustained over all non-exercise follow-up time points (i.e., 9 months and 12 months). Leg lean tissue cross sectional area, however, remained unchanged immediately following the MCEFRP but decreased (1%) in the non-exercise follow-up period. Previously eccentric resistance exercise, when compared to more traditional resistance exercise, has demonstrated greater effects and/or superior mobility, balance and muscle responses in high fall risk older adults [15]. The comparison of the current large randomized trial to this small non-randomized pilot study is difficult as a different eccentric resistance exercise device was used and dissimilar testing modes were employed. A more recent randomized study [19], however, may be a more appropriate comparator as similar muscle and mobility outcomes were reported when using the identical eccentric device and protocol for 3 months of resistance exercise with older survivors of cancer (90% having 8+ years of survival since their breast, prostate or colorectal cancer diagnosis). Those survivors of cancer who performed eccentric resistance exercise were comparable to the high fall risk individuals who participated in the MCEFRP as they were equivalent in age (75 years), gender distribution (>60% female), BMI (28 kg*m^−2^), mobility level (pre-exercise 6 MW distance of 417 m) and were characterized by a similar suite and number of comorbidities. With that, this report of a sample of high fall risk older adults who participated in a pragmatic fall prevention trial, highlighted by rigorous surveillance of fall events, represents similar usefulness of eccentric resistance exercise and the equivalence of either RENEW or TRAD as part of a MCEFRP. We conclude the community dwelling older adults did improve their annual fall event rate as 100% of participants had fallen in the year prior to the MCEFRP, but during the year following enrollment <50% of participants experienced a day when a fall or near fall occurred.

It can be challenging when interpreting the results from this large randomized trial when a no-intervention control group was not included, however, this study was not designed to determine the effectiveness of a MCEFRP. More than 150 clinical trials have evaluated exercise in the prevention of falls [21]. Nor was this study designed to determine the effects of resistance exercise alone on the risk of falls. This too has been dissected in at least eight previous studies in samples with the average age greater than 75 years [54–61]. Rather, this study was designed as a larger extension to previous smaller eccentric trials that suggested the effect of RENEW might be superior to TRAD. We have clearly concluded that an effective MCEFRP can use either RENEW or TRAD as its mode of strength training. Less clear are how to interpret the respective mobility, muscle and balance responses to the MCEFRP as they were variable in both the RENEW and TRAD groups making it difficult to decipher whether one of these risk factors might differentially influence the number of days a high fall risk participant survived without a fall or fall event. Finally, despite rigorous accounting of fall and near fall events (and 3 months of extensive training of the participants during the MCEFRP on what constitutes a fall or near fall event), the latter can be subjective. Therefore, near falls may have been either over- or under-represented in our results. We included near falls in our study design since it has been used as an outcome previously and prior research has indicated older adults can distinguish near falls [62].

There are several strengths to this test of RENEW versus TRAD as part of a MCEFRP that collectively make it unique. The trial design met the recent recommendations stemming from a meta-analysis [23] for fall prevention practices using exercise in community dwelling older adults. The recruitment strategy was successful in targeting a large number of well-characterized, community-ambulating older adults who were clearly at-risk for falling having experienced one or more falls in the previous year and averaging more than five comorbidities. The MCEFRP was in a group exercise setting for 3 h per week and included two approaches to strength training coupled with high challenges to balance. Adherence and participation rates were high and both performance and self-report outcomes were assessed. Finally, this study followed the consensus recommendations for the acute monitoring of fall events during and immediately following MCEFRP. Further, the monthly fall surveillance approach over the 1 year follow-up is a strength as collectively these fall event tracking approaches are employed in less than 50% of previous multicomponent fall prevention trials.

## Conclusion

The purpose of this study was to test if RENEW or TRAD had differential effects on both fall risks and fall events in a high fall risk group of older adults. We conclude the two modes of resistance exercise incorporated into a MCEFRP had identical effects.

## Abbreviations

6 MW: Six-minute walk test
ABC: Activities specific balance confidence scale
BMI: Body mass index
MCEFRP: Multi-component exercise fall reduction program
MRI: Magnetic resonance imaging
RENEW: Resistance exercise via negative, eccentrically-induced, work
TRAD: Traditional resistance exercise
